# Expression, prognosis value, and immune infiltration of lncRNA ASB16-AS1 identified by pan-cancer analysis

**DOI:** 10.1080/21655979.2021.1996054

**Published:** 2021-12-23

**Authors:** Linyong Wu, Wei Liao, Xiaodong Wang, Yujia Zhao, Jinshu Pang, Yuji Chen, Hong Yang, Yun He

**Affiliations:** Department of Medical Ultrasound, The First Affiliated Hospital of Guangxi Medical University, Nanning, P. R. China

**Keywords:** Pan-cancer, Long non-coding RNA ASB16-AS1, Prognosis, Immune infiltration, Molecular characteristics

## Abstract

Long non-coding RNA known as ASB16 antisense RNA1 (ASB16-AS1) has been proven to be an oncogene, and the relationship between ASB16-AS1 and immunity is still under studied. This study aims to explore the expression and prognostic potential of ASB16-AS1, and to visualize the relationship between ASB16-AS1 expression and immune infiltration in pan-cancer analysis. We clarified ASB16-AS1 expression patterns and its relationship with prognosis through multi-platform and multi-database sources. We also verified the function of ASB16-AS1 in liver hepatocellular carcinoma (LIHC). A  variety of immune cell content evaluation methods were used to mutually verify the correlation between ASB16-AS1 and immune infiltration. Finally, the relationships between ASB16-AS1 and molecular characteristics were further explored. In terms of comprehensive analysis, compared with non-tumor tissues, ASB16-AS1 was highly expressed in tumor tissues, and indicated the value of poor prognosis in multiple cancer types. Functional assays, such as counting kit-8 assay, transwell assay and scratch-wound assay verified that high ASB16-AS1 expression promoted tumor progression in LIHC. ASB16-AS1 was positively correlated with B cells, T cells CD4+ and T cells CD8+ in most cancer types, and negatively correlated with macrophages, dendritic cells and neutrophils in some cancer types. In addition, there were different interaction modes between ASB16-AS1 and molecular features, such as the relationship with oncogenic signaling pathways, showing that the high ASB16-AS1 expression was related to alterations in oncogenic signaling pathways. Our study emphasizes that ASB16-AS1 is a potential pan-cancer prognostic marker, whichs is associated with the immune infiltration in multiple cancer types.

## Introduction

Tumor immunology and immunotherapy have become holty pursued topics in cancer research [1]. The purpose of immunotherapy is to activate the immune system so that it recognizes and destroys cancer cells, which provides many patients with deep, long-term relief and potential cure prospects. However, immunotherapy still has such as high recurrence rate, toxic effects, and immune escape [2], indicating that there are still major challenges for immunotherapy to be considered fully pre-clinical. Long non-coding RNAs (lncRNAs) are RNAs that do not have protein coding ability and are longer than 200 nucleotides [3]. These lncRNAs have become regulators of almost all biological functions. They can interact with RNA, DNA or protein to promote or inhibit the expression of protein-coding genes [4]. In recent years, lncRNAs have been shown to be important molecules involved in the immune landscape [5]. For example, lncRNAs have been shown to regulate the expression of immune checkpoint molecules in cancers [6], while lncRNA GATA3-AS1 promoted triple-negative breast cancer progression and immune evasion by stabilizing programmed cell death protein-1 (PD-L1) and degrading GATA3 protein [7]. Furthermore, lncRNA SATB2-AS1 affected the microenvironmental changes of colorectal cancer tumor immune cells by regulating SATB2 [8]. However, the relationship between numerous lncRNAs and immunity still need further exploration and verification.

In recent years, the integration of genomics, transcriptomics, and clinicopathological parameters through multi-platform and multi-omics perspectives has further clarified the landscape of molecular characterization in a variety of tumors, and promoted the visual analysis of the role of molecules [9]. LncRNA ASB16 antisense RNA 1 (ASB16-AS1) is located on the 17th chromosome. It has been proven to be an oncogene that promotes the occurrence and development of multiple cancer types. For example, ASB16-AS1 promoted the proliferation and invasion of gastric cancer by regulating the expression level of TRIM37 [10]; Up-regulation of ASB16-AS1 expression promoted the proliferation and inhibited apoptosis in non-small cell lung cancer by Wnt signaling pathway [11]; ASB16-AS1 regulated the up-regulation of HDGF expression to promote the malignant phenotype of osteosarcoma by sponging miR-760 [12]. However, the role of ASB16-AS1 in cancers is still in the preliminary stage of exploration, and the mechanism of ASB16-AS1 in tumor immunology is still at a blank stage.

In this study, a pan-cancer analysis of the transcriptional landscape of ASB16-AS1 was developed to further clarify its functional role of cancers. We comprehensively identified the relationship between ASB16-AS1 expression and cancer development/patient prognosis in 33 cancer types, and it was verified that ASB16-AS1 promoted the proliferation and invasion in hepatocellular carcinoma (LIHC) through related assays. In addition, the relationships between ASB16-AS1 expression and immune infiltration, molecular characteristics (immune checkpoints, tumor mutation burden, microsatellite instability, oncogenic signaling pathways, and radiomics). Our results revealed the possible role of ASB16-AS1 in cancers, indicating that ASB16-AS1 was a potential prognostic biomarker, whichs is associated with the immune infiltration in multiple cancer types.

## Materials and methods

### Database retrieval

The cancer genome atlas (TCGA), Genotype Tissue Expression (GTEx), Gene Expression Omnibus (GEO), ArrayExpress and Sequence Read Archive (SRA) were all freely accessible and downloadable public databases, which provided gene expression data sets and phenotype data of more than 10,000 tumor samples. The RNA-seq data of GTEx and TCGA databases were obtained from the UCSC Xena data center (http://xena.ucsc.edu/) in FPKM format.

### Transcriptome landscape of lncRNA ASB16-AS1 in different cancer types

ASB16-AS1 expression level in different cancer types were identified by *Mann-Whitney U* test. In view of the differences between different data processing methods, the SangerBox online platform (http://sangerbox.com/) was used to analyze the differences in TPM data processing. In response to the problem of fewer non-cancer samples in the TCGA database, the non-cancer tissue datas in the GTEx database were integrated into the data in the TCGA database on the SangeBox analysis platform, in order to further expand the sample cross-validation of ASB16-AS1expression level using the same method.

### Verification of the transcriptional landscape of lncRNA ASB16-AS1 in liver hepatocellular carcinoma

To further verify ASB16-AS1 expression in LIHC, we integrated and analyzed ASB16-AS1 expression in multiple databases (GEO, ArrayExpress, and SRA) with the keyword ‘liver’. The inclusion criteria are: (1) the data collection method was RNA microarray and RNA sequencing; (2) from human tissue or blood; (3) the number of tumor or non-tumor tissues was ≥3. In addition, the pooled standardized mean difference (SMD) of evidence-based evaluation (fixed and random-effects models) was calculated by Stata software for the ASB16-AS1 high expression. The random-effects model would be considered for the presence of significant heterogeneity (I^2^ > 50%), while the fixed-effects model would be used when low heterogeneity was detected. The pooled SMD and its lower 95% confidence interval (CI) of greater than 0 indicated high ASB16-AS1 expression compared with non-tumor tissues in LIHC [13].

### Verification of the function of lncRNA ASB16-AS in liver hepatocellular carcinoma

#### Cell culture

Five hepatocellular carcinoma (HCC) cell lines (HepG3B, Huh-7, THLE-3, MHCC97L and MHCC97H) were purchased from Shanghai Cell Bank of Chinese Academy of Sciences, and culture conditions were as follows: 10% fetal bovine serum and RPMI1640/DMEM mixed medium (1:1), 37 °C, and 5% CO2 incubator.

#### Quantitative real-time PCR

TRIzol reagent (Invitrogen, USA) was used to isolate total RNA, and RevertAid Reverse Transcriptase (Thermo, USA) was used to synthesize total cDNA. SYBR® Green Realtime PCR Master Mix (GENVIEW, USA) was used for real-time PCR. Standardization was based on Glyceraldehyde-3-phosphate dehydrogenase. The primers of ASB16-AS1 was as follows: forward: GTGACACTCCCTTGCCTTTC, reverse: GCAGCCACTAACTTGCTGTG. 2-ΔΔCt method was used to calculate the relative expression level.

#### Cell transfection

ASB16-AS1 small interfering RNA (siRNA) and negative control (NC) siRNA were synthesized by Huzhou Hippo Biotechnology Co., Ltd. (Zhejiang, China), and the transfection procedure of this study was used by following the manufacturer’s recommendations:

ASB16-AS1 si-1: GCUACUCACCUCGUUCUACUUTT;

ASB16-AS1 si-2: GUGAGAACCACUGGCCUAGUATT;

ASB16-AS1 si-3: GGUAAUUCAUUGAGGCACUCUTT.

Total RNA was extracted 24 hours after transfection, and RT-qPCR was used to evaluate the effect of transfection.

#### Cell proliferation assay

CCK8 assay was for detecting cell proliferation ability after transfection. The HCC cells were seeded into a 96-well plate and cultured for 96 h. Next, CCK8 solution was added at 24

#### Cell invasion and migration assay

Transwell assay and Wound scratch assay were used to evaluate the invasion and migration ability of cells after transfection.

#### Cell apoptosis assay

Annexin V-FITC/PI (Biolegend, USA) double-stained cell apoptosis detection kit was used for fluorescent labeling, and flow cytometry (CytoFLEX, BECKMAN) was used to evaluate cell apoptosis.

#### Identification of lncRNA ASB16-AS1 in different stages

To verify the expressionl of ASB16-AS1 in tumor progression, Gene Expression Profilling Interactive Analysis (GEPIA) online analysis platform was for comparing the overall expression differences of all stages (*Kruskal-Wallis H test*). Then, the ‘limma’ package was for comparing the expression differences of tumors in different stages.

### Identification of the prognostic potential of lncRNA ASB16-AS1

The Kaplan-Meier analysis was for evaluating the prognostic potential of ASB16-AS1 in different cancers, including overall survival (OS) and progression-free interval (PFI). According to the median of gene expression, the different types of cancer data were identified as high expression and low expression groups, and then Log-rank test was for testing whether they were statistically significant. In addition, the prognostic potential of ASB16-AS1 was further analyzed on Gene Expression GEPIA online analysis platform for cross-validation.

### Identification of the relationship between lncRNA ASB16-AS1 and immune infiltration

The estimate R package was used to estimate the tumor microenvironment (immune cell, stromal cell and tumor purity) in each cancer sample [14]. The TIMER (version 2.0) online website (http://timer.cistrome.org/) was used to obtain the immune cell contents assessed by different methods (TIMER, CIBERSORT, quanTIseq, xCell, MCP-counter and EPIC) by the immuneeconv R package analysis [15]. 47 immune checkpoints gene expression in all cancer samples were obtained. Tumor mutation burden (TMB) and microsatellite instability (MSI) are potential markers for evaluating the efficacy of immune checkpoint inhibitor therapy [16,17]. TMB referred to the number of mutations per million bases in each sample. The perl software was used to calculate the TMB value of all samples. MSI is defined as a phenomenon in which deletion or insertion mutations cause changes in the length of tumor microsatellites during DNA replication. The MSI values of all samples were obtained from previous studies.

### Identification of the relationship between lncRNA ASB16-AS1 and oncogenic signal pathways

Gene Set Enrichment Analysis (GSEA) was for detecting the abundant signal pathways between the higher and lower ASB16-AS1 groups in each cancer type. In the R language environment, ‘c5.all.v7.1.symbols.gmt’ was used as a reference gene set, and clusterProfiler, org.Hs.eg.db and enrichplot packages were used to discover gene ontology (GO) affected terms by ASB16-AS1 [18]. Signal pathways standardized enrichment score (NES) > 1.0, and with standardized *p* < 0.05 were considered statistically significant.

### Identification of the relationship between lncRNA ASB16-AS1 and radiomics

As a noninvasive technology, radiomics has now become the cornerstone of precision medicine. The radiomics features can be used to analyze the occurrence or progression of cancer through exploration and discovery [19]. Changes in cancers are also accompanied by changes in genes. Whether there are certain parallel relationship between radiomics features and gene expression is worthy of discussion. Therefore, the relationship between ASB16-AS1 and computed tomography-radiomics features (HCC, samples  =36, KIRC, samples =171) were identified. 7 major types of radiomics features were analyzed: 122 original, 48 intraperinodular textural transition (Ipris), 1170 co-occurrence of local anisotropic gradient orientations (CoLIAGe), 432 wavelets + local binary pattern (LBP), 1080 Gabors, 80 congruency-based local binary pattern (PLBP) and 60 wavelet-based improved local binary pattern (WILBP) features. Different radiomics features represent different. For example, Ipris features capture the transition in textural appearance going from the inside to the outside of the nodule; CoLIAGe features distinguisheed disease phenotypes with similar morphological appearance.

### Statistical analysis

SPSS (version 23.0), Stata (version 2.0), and R (version 3.6) softwares were used for statistical analysis and graphing. ASB16-AS1 expression level in different types were identified by *Mann-Whitney U test*. The comprehensive analysis of multiple databases with the median value of all samples was used by Stata software. Functional asssys were analyzed by *t test*. Spearman correlation test was used to identify the interaction potential of ASB16-AS1 with tumor microenvironment, immune cells, immune checkpoint genes, TMB, MSI, and radiomics features. All statistical analysis took *p* <0.05 as the standard of statistical significance (*p* <0.05 was represented by ‘*’, *p* <0.01 was represented by ‘**’, *p* <0.001 was represented by ‘***’).

## Results

### Transcriptome landscape of lncRNA ASB16-AS1 and verification analysis in liver hepatocellular carcinoma

**Figure 1.**
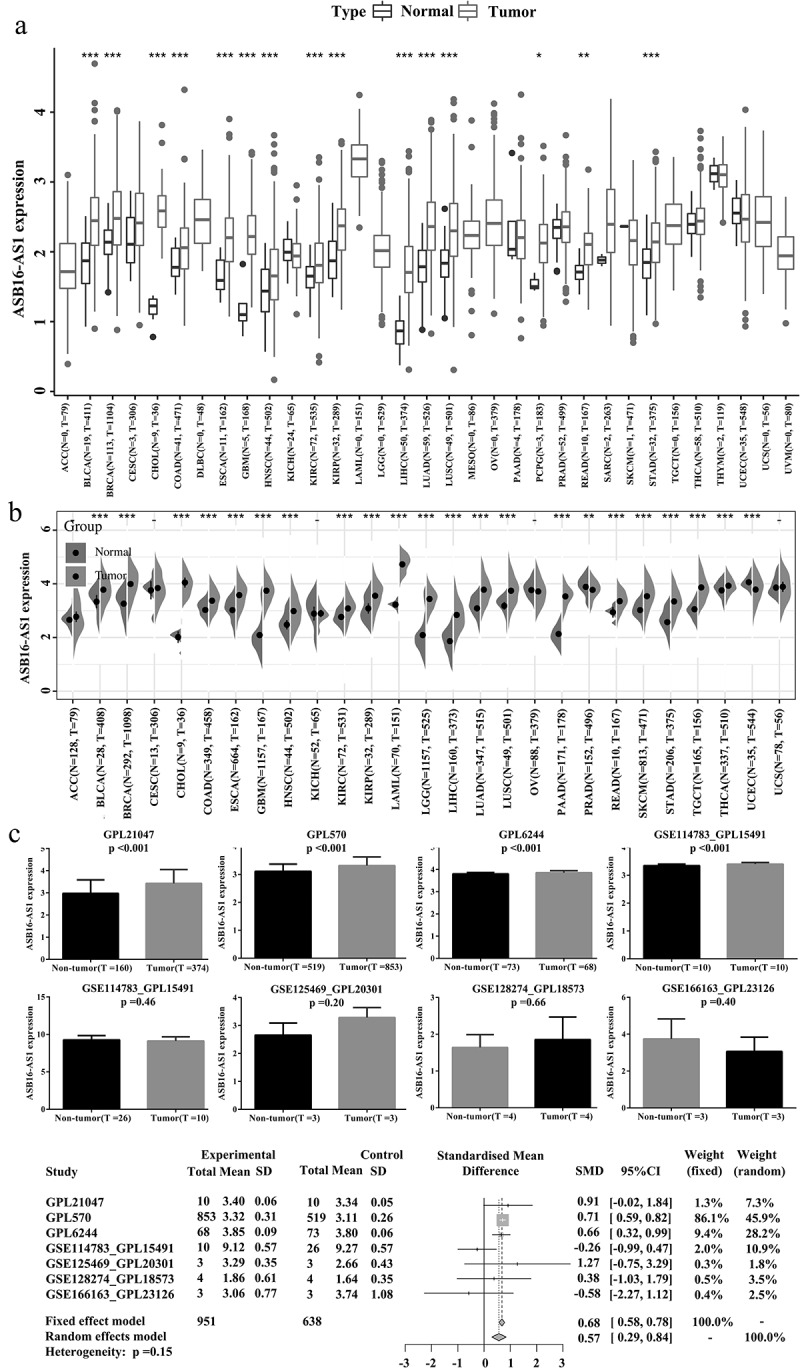
**Transcriptome landscape of lncRNA ASB16-AS1 and verification analysis in**LIHC. (a) The lncRNA expression of ASB16-AS1 between tumor tissues and non-tumor tissues in 33 cancer types of TCGA. (b) ASB16-AS1 expression between tumor and non-tumor tissues of TCGA and GETx (TPM). (c) 7 platform data of GEO database verifred the high ASB16-AS1 expression in LIHC. Except GSE166163_GPL23126, 6 platform data displayed that ASB16-AS1 was highly expressed in LIHC samples, and there were significant differences in 3 platforms (Mann-Whitney U test, GPL570, *p* <0.001; GPL21047, *p* =0.019 and GPL6244, *p* <0.001). The random effect model evaluation model showed that compared with non-tumor samples, ASB16-AS1 was highly expressed in LIHC samples, with an SMD of 0.57 (95% CI: 0.29–0.84)

The theme of this studywas to explore the clinical significance of lncRNAASB16-AS1and its various molecular association analysis in cancers, aiming to clarify the potential role of ASB16-AS1 in tumorigenesis, development and treatment-related. Based on the TCGA database, we discussed ASB16-AS expression, prognostic correlation, and correlation with various molecular characteristics. In addition, we conducted preliminary verification in LIHC.Transcriptome landscape of lncRNA ASB16-AS1 and verification analysis in LIHC”. Among the 33 cancer types in the TCGA database, ASB16-AS1 was differentially expressed in 15 cancer types, and also was differentially highly expressed, including bladder urothelial carcinoma (BLCA), breast invasive carcinoma (BRCA), cholangiocarcinoma (CHOL), colon adenocarcinoma (COAD), esophageal carcinoma (ESCA), glioblastoma (GBM), head and neck squamous cell carcinoma (HNSC), kidney renal clear cell carcinoma (KIRC), kidney renal papillary cell carcinoma (KIRP), LIHC, lung adenocarcinoma (LUAD), lung squamous cell carcinoma (LUSC), pheochromocytoma and paraganglioma (PCPG), rectum adenocarcinoma (READ), stomach adenocarcinoma (STAD) (Figure 1a). When analyzed with TPM data, except for PCPG, ASB16-AS1 was differentially highly expressed in the same 14 cancer types. The expanded sample analysis results of the SangeBox analysis platform showed that after combining with the GTEx non-cancer tissue database, ASB16-AS1 was differentially expressed in 22 cancer types, of which 20 cancer types were highly expressed: BLCA, BRCA, CHOL, COAD, ESCA, GBM, HNSC, KIRC, KIRP, acute myeloid leukemia (LAML), brain lower grade glioma (LGG), LIHC, LUAD, LUSC, pancreatic adenocarcinoma (PAAD), READ, skin cutaneous melanoma (SKCM), STAD, testicular germ cell tumors (TGCT), thyroid carcinoma (THCA). and 2 cancer types were low in expression: prostate adenocarcinoma (PRAD) and uterine corpus endometrial carcinoma (UCEC) (Figure 1b). In general, the high expression of ASB16-AS1 was closely related to the occurrence of multiple cancer types.

The datasets of 7 GEO platforms were for evaluating ASB16-AS1 expression in LIHC: GPL570, GPL21047, GPL6244, GSE114783_GPL15191, GSE128274_GPL18573, GSE125469_GPL20301 and GSE166163_GPL23126. Except GSE166163_GPL23126, 6 platforms indicated that ASB16-AS1 was highly expressed in LIHC samples, and there were significant differences in 3 platforms (*Mann-Whitney U test*, GPL570, *p* <0.001; GPL21047, *p* =0.019 and GPL6244, *p* <0.001). In addition, all available samples (951 cancer samples, 638 non-cancer samples) were combined to obtain a reliable estimate of ASB16-AS1 expression. The random effect model evaluation model showed that compared with non-tumor samples, ASB16-AS1 was highly expressed in LIHC samples, with an SMD of 0.57 (95% CI: 0.29–0.84) (Figure 1c). The big data samples further verified that ASB16-AS1 was highly expressed in LIHC and was closely related to the occurrence of cancers.

### Verification of the function of lncRNA ASB16-AS in liver hepatocellular carcinoma

**Figure 2.**
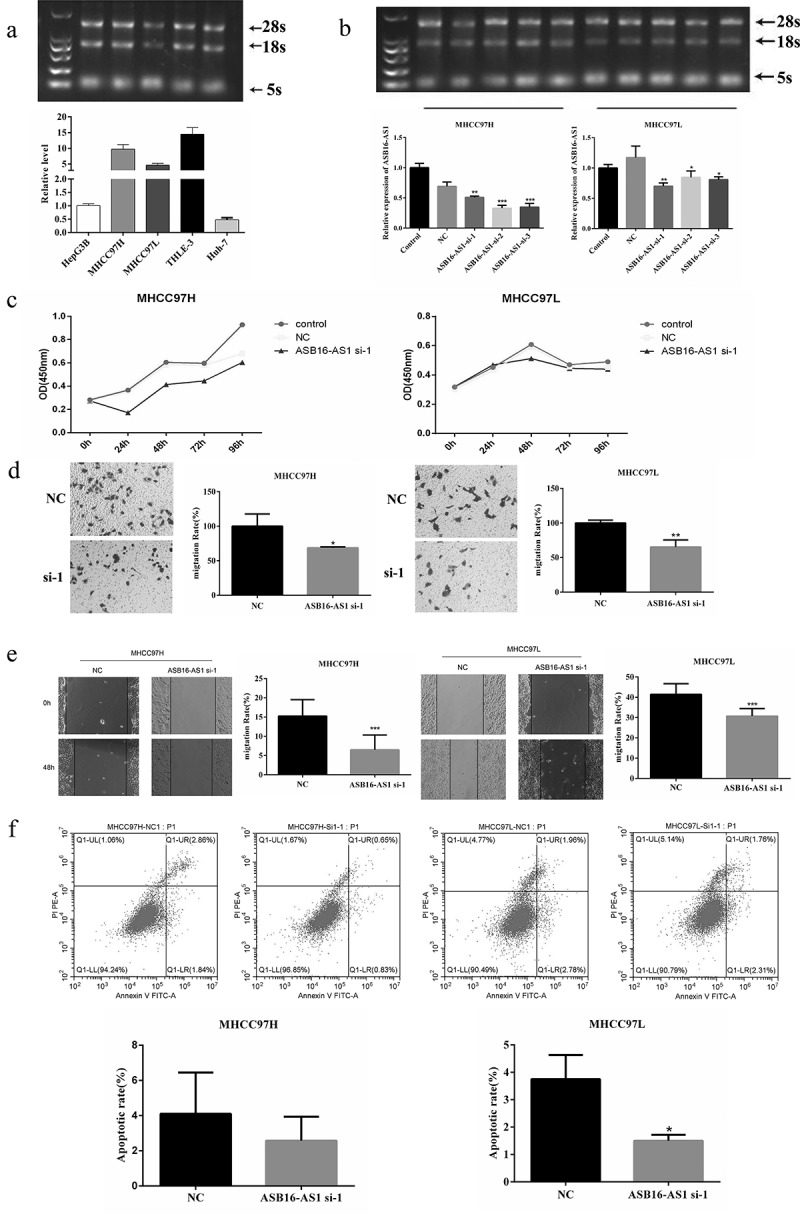
**ASB16-AS1 up-regulation promoted LIHC proliferation, migration, and invasion**. (a) The expression pattern of ASB16-AS1 in 5 HCC cell lines. (b) The ASB16-AS1 knockdown effect caused by siRNA was evaluated in MHCC97H and MHCC97L cells. (c) CCK8 was used to evaluate the proliferation ability of HCC cell lines. (d-e) Scratch-wound assay and transwell assay were used to evaluate the proliferation, invasion and migration capabilities of HCC cell lines. (f) Flow Cytometry was used to assess the level of apoptosis in HCC cell lines

In order to explore the function of ASB16-AS1 in LIHC, vitro cell assays were performed. ASB16-AS1 up-regulated in 5 HCC cell lines, and was significantly up-regulated in MHCC97H, MHCC97L and THLE-3 cell lines compared to other cell lines (Figure 2a). Then, in order to form a comparison, MHCC97H and MHCC97L cell lines were entered the next assays. ASB16-AS1 expression was knocked down by siRNA. Compared with NC, ASB16-AS1 expression in two cell lines were significantly reduced (Figure 2b). The knockdown effect of ASB16-AS1-si-2 was the strongest in MHCC97H cell, and the knockdown effect of ASB16-AS1-si-1 was the strongest in MHCC97L cell. Next, ASB16-AS1-si-1 was confirmed for further assays. CCK8 assay showed that ASB16-AS1 down-regulation weakened the proliferation ability of MHCC97H and MHCC97L cells (Figure 2c). Compared with the NC group, the transwell assay showed that the knockdown of ASB16-AS1 attenuated the migration and invasion ability of MHCC97H and MHCC97L cells (Figure 2d). In addition,  scratch-wound assay also confirmed that knockdown of ASB16-AS1 weakened the migration ability of cells (Figure 2e). According to flow cytometry analysis, in MHCC97H and MHCC97HL cells, knockdown of ASB16-AS1 could reduce cell apoptosis to a certain extent. In MHCC97L cells, a comparison of NC and ASB16-AS1 si-1 groups was found to be statistically significant (figure 2f). In general, the up-regulation of ASB16-AS1 expression promoted the proliferation and invasion of HCC, which may lead to tumor progression.

### Identification of lncRNA ASB16-AS1 in different stages

**Figure 3.**
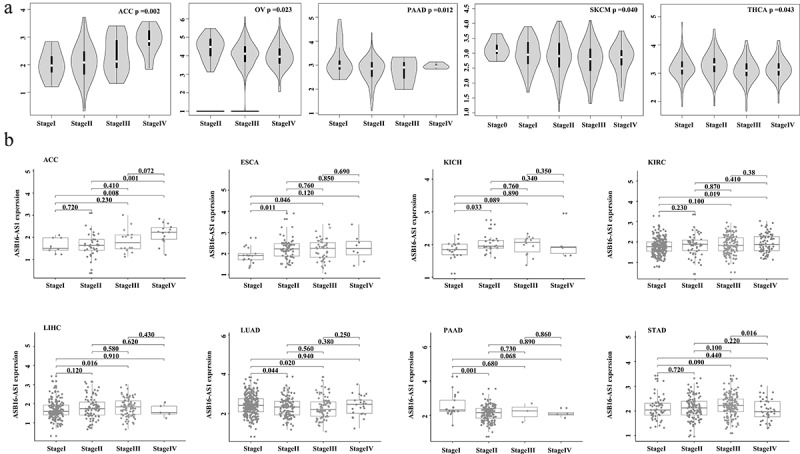
**Identification of lncRNA ASB16-AS1 in different stages**. (a) ASB16-AS1 was differentially expressed in the overall stages of 5 cancer types by GEPIA analysis. (b) In the comparison of different stages, ASB16-AS1 was differentially expressed in 8 cancer types

The ASB16-AS1 expression level increased with tumor stage, which confirmed that ASB16-AS1 promoted tumor development. The results showed that in the 5 cancer types—adrenocortical carcinoma (ACC, *p* =0.002; ovarian serous cystadenocarcinoma (OV), *p* =0.023; PAAD, *p* =0.012; SKCM, *p* =0.040 and THCA, *p* =0.043)—ASB16-AS1 was differentially expressed in the overall stages (Figure 3a). In the comparison of different stages, ASB16-AS1 was differentially expressed in 8 cancer types [(ACC, I vs IV, *p* =0.008; II vs IV, *p* =0.001); (ESCA, I vs II, *p* =0.011; I vs III, *p* =0.046); (kidney chromophobe, KICH, I vs II, *p* =0.033); (KIRC, I vs IV, *p* =0.019); (LIHC, I vs III, *p* =0.016); (LUAD, I vs II, *p* =0.044; I vs III, *p* =0.020); (PAAD, I vs II, *p* =0.009); (STAD, III vs IV, *p* =0.016)]. In general, the increased expression of ASB16-AS1 has the potential to promote the development of cancer types to a certain extent (Figure 3b).

### Identification of the prognostic potential of lncRNA ASB16-AS1

**Figure 4.**
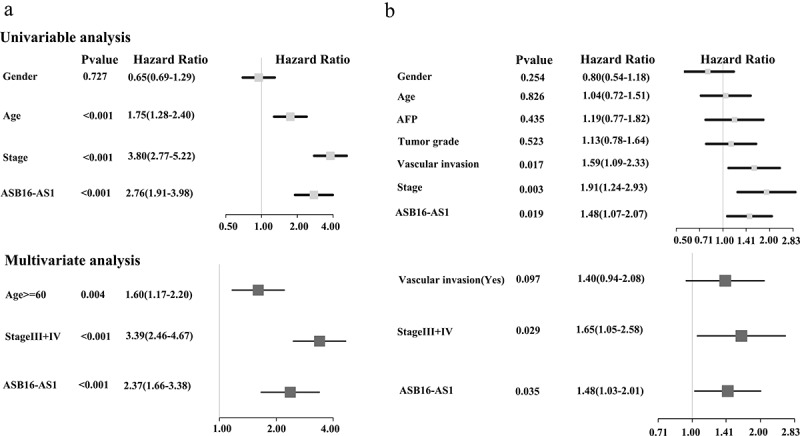
**Identification of the prognostic potential of ASB16-AS1 in KIRC and LIHC by multivariate Cox regression analysis**. (a) ASB16-AS1 expression was a high risk factor for shorter OS in KIRC. (b) ASB16-AS1 expression was a high risk factor for shorter PFS in LIHC

The Kaplan-Meier analysis showed that in 3 cancer types—ACC, *p* =0.009; COAD, *p* =0.014; and KIRC, *p* <0.001)—high ASB16-AS1 expression was associated with worse OS; in the other 3 cancers—BLCA, *p* =0.003; mesothelioma (MESO), *p* =0.040; and PAAD, *p* =0.043—high ASB16-AS1 expression represented better OS. In addition, among the 3 cancer types—ACC, *p* =0.009; LIHC, *p* =0.019; and PRAD, *p* =0.016—high ASB16-AS1 expression represented worse PFS; only one cancer type—PAAD, *p* <0.001) represented better PFS. For 6 cancer types with high expression and poor prognosis, multivariate Cox regression analysis with clinical factors was performed. The results showed that only in KIRC ASB16-AS1 was a high-risk factors for OS (HR = 2.37, *p* <0.001) (Figure 4a); only in LIHC, ASB16-AS1 was high-risk factors for PFS (HR = 1.4, *p* =0.040) (Figure 4b). GEPIA online analysis showed that high ASB16-AS1 expression represented worse OS among the 3 cancer types: COAD, *p* =0.028; GBM, *p* =0.035; and KIRC, *p* =0.015. Only one cancer type represented worse PFS (LIHC, *p* =0.001). In general, ASB16-AS1 high expression was closely related to the prognosis of multiple cancer types, especially LIHC.

### Identification of the relationship between lncRNA ASB16-AS1 and immune infiltration

**Figure 5.**
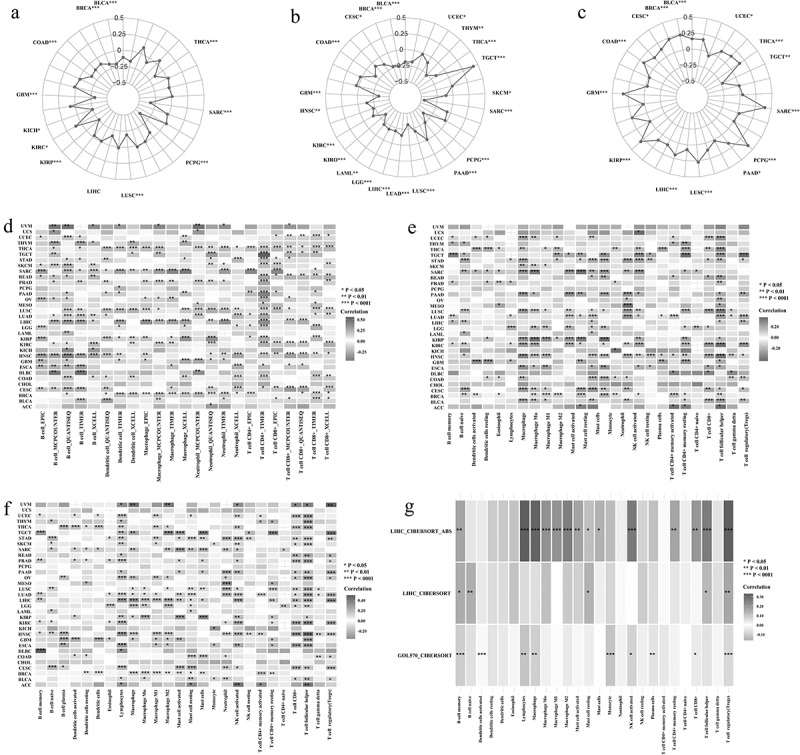
**Identification of the relationship between lncRNA ASB16-AS1 and immune infiltration**. (a-c) The relationship between ASB16-AS1 expression and tumor microenvironment. a, ImmuneScores; b, StromalScores; and c, TumorPurityScores. (d) Identification of the relationship between ASB16-AS1 and 6 types of immune cells based on data from multiple immune cell evaluation methods. (e) Identification of the relationship between ASB16-AS1 and 22 types of immune cells based on CIBERSORT data. (f) CIBERSORT-ABS data further validated the results of the above 22 types of immune cells. (g) GEO’s GPL570 platform data verified the relationship between ASB16-AS1 and 22 types of immune cells in LIHC

The tumor microenvironment in different parts of the tumor has been confirmed to have different physiological and pathological significance. Therefore, we have identified the relationship between ASB16-AS1 and tumor microenvironment (ImmuneScores, StromalScores and TumorPurityScores) (Figure 5a-c). There were significant correlations between ASB16-AS1 expression and ImmuneScores of 12 cancer types, StromalScores of 20 cancer types, and TumorPurityScores of 14 cancer types. Regarding immune cell score, except for the positive correlation in KIRC (r =0.09, *p* =0.030), there were negative correlation in the other 11 kinds of cancers, among which strongest was in PCPG (r =−0.41, *p* <0.001) and the most significant in BRCA (r = −0.17, *p* <0.001). For stromal cell score, only positive correlation was found in TGCT (r = 0.42, *p* <0.001). There werenegative correlations in other cancer types, among which the correlation in SARC (r =−0.45, *p* <0.001) was the strongest and the most significant. In the correlation analysis of TumorPurityScores, only TGCT (r =−0.23, *p* =0.004) showed negative correlation, while the other 13 showed positive correlation, among which the correlation was the strongest in PCPG (r =0.40, *p* <0.001). In addition, the liver cancer chip (GSE114783_GPL15491) further verified that ASB16-AS1 was negatively correlated with ImmuneScores (pearson test, r =−0.76, *p* =0.009), StromalScores (pearson test, r =−0.54, *p* =0.100) and TumorPurityScores (pearson test, r =0.78, *p* =0.006). In general, ASB16-AS1 expressionis was negatively correlated with tumor microenvironment in multiple cancer types.

Tumor is a complex environment composed of transformed cells, stroma and immune infiltration. Tumor infiltrating cells can show anti-tumor or tumor-promoting effects, depending on the type of cancer or tumor model. Through a variety of immune cell content assessment methods, we had compared and summarized the relationship between ASB16-AS1 and the six immune cells (B cells, dendritic cells, macrophages, neutrophils, T cells CD4+ and T cells CD8+). The results showed that ASB16-AS1 was positively correlated with B cells, T cells CD4+ and T cells CD8+ in most cancer types, and negatively correlated with dendritic celsl, macrophages and neutrophils in some cancer types (>14), especially neutrophils (Figure 5d). The results of the CIBERSORT evaluation showed that ASB16-AS1 was significantly positively correlated with lymphocytes, and significantly negatively correlated with macrophages, neutrophils and eosinophils. ASB16-AS1 was positively correlated with T cells regulatory (Tregs) in most cancer types, especially TGCT (r =0.35, *p* <0.001) and KIRC (r =0.25, *p* <0.001). We were concerned about the relationship between AB16-AS1 and T cells follicular helper cells. ASB16-AS1 was positively correlated with T cells follicular helper cells in 30 cancer types, especially ACC (r =0.35, *p* =0.026), HNSC (r =0.34, *p* <0.001) and KIRC (r =0.34, *p* <0.001) (Figure 5e). The above results were verified again by the CIBERSORT-ABS evaluation method (figure 5f). In addition, through the GPL570 external chip of LIHC, we verified that some immune cells had the same expression pattern as the TCGA results (Figure 5g). In general, in LIHC, ASB16-AS1 was positively correlated with most immune cells, especially macrophages (r =0.32, *p* <0.001), T cells regulatory (Tregs) (r =0.33, *p* <0.001), lymphocytes (r =0.33, *p* <0.001), which showed a strong correlation and only negatively correlated with B cells naive, mast cells, monocytes, NK cells resting and T cells gamma delta. It must be noted that the different functions of different breast cancer subtypes correspond to the relationship between different immune cells and ASB16-AS1. In BRCA-basal, ASB16-AS1 was positively correlated with T cells CD4+ memory activated, macrophages M1, macrophages M0, T cells CD4+ memory resting, dendritic cells resting, and was negatively correlated with NK cells activated, monocytes, B cells naive, T cells follicular helper, mast cells activated.

**Figure 6.**
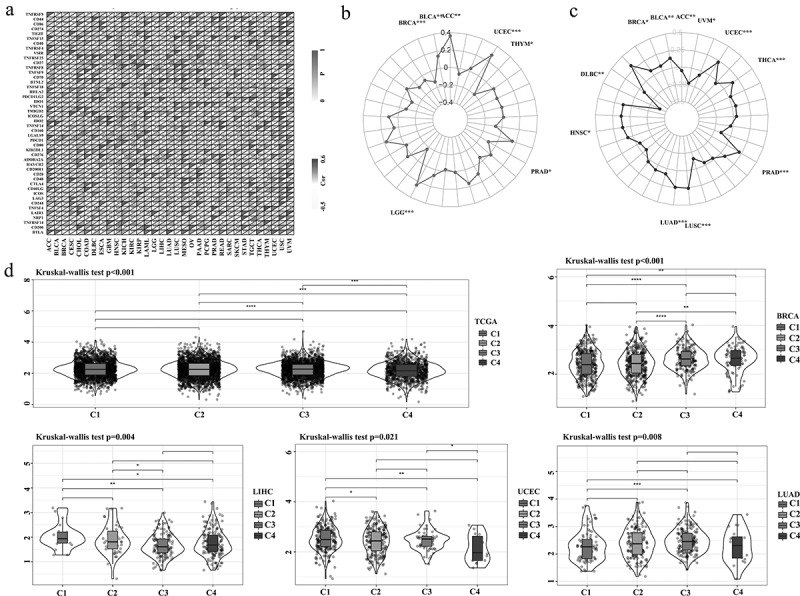
**Identification of the relationship between ASB16-AS1 and potential prognostic biomarkers of immunotherapy**. The relationships between ASB16-AS1 and immune checkpoint genes (a), TMB (b), MSI(c). (d) ASDB16-AS1 expression in different immune subtypes in BRCA (*p* <0.001), LIHC (*p* =0.001), LUAD (*p* =0.008), and UCEC (*p* =0.021)

Checkpoint inhibitor blockade immunotherapy has a new wave of anti-cancer research. The relationships between ASB16-AS1 and 47 common immune checkpoint genes was identified. ASB16-AS1 had significant correlation with most of the immune checkpoint genes in BLCA, BRCA, GBM, HNSC, KIRC, KIRP, LGG, LUAD, READ, SKCM, and TGCT. We focused on the relationship between ASB16-AS1 and CD274 (PD-L1). AB16-AS1 was positively correlated with 4 cancer types, and negatively correlated with 8 cancer types, especially KIRP (r =−0.32, *p* <0.001) and MESO (r =−0.27, *p* =0.010) (Figure 6a).

TMB and MSI are considered as new biological markers for evaluating the efficacy of immunotherapy. ASB16-AS1 expression were positively correlated with TMB in 4 cancer types: ACC (r = 0.36, p =0.001), UCEC (r =0.28, p <0.001), LGG (r =0.24, p <0.001), BLCA (r =0.14, p =0.004), and negatively correlated with 3 cancer types: THYM (r = −0.20, p =0.033), BRCA (r =−0.13, p <0.001), LGG (r =−0.10, p =0.040) (Figure 6b). ASB16-AS1 expression were positively correlated with MSI in 8 cancer types: LUSC (r =0.26, *p* <0.001), LUAD (r =0.24, *p* <0.001), UCEC (r =0.22, *p* <0.001), PRAD (r =0.21, *p* <0.001), THCA (r =0.15, *p* =0.001), BLCA (r =0.14, *p* =0.004), HNSC (r =0.11, *p* =0.011), BRCA (r =0.07, *p* =0.018), and negatively correlated with 2 cancer types: DLBC (r =−0.40, *p* =0.005) and UVM (r =−0.22, *p* =0.047) (Figure 6c).

Based on the molecular typing of immune subtypes [20], ASB16-AS1 expression in different subtypes were analyzed. In all cancer samples, ASB18-AS1 expression had an overall significant difference among the four immune subtypes [C1 (wound healing), C2 (IFN-γ dominant), C3 (inflammatory), C4 (lymphocyte deplete)] (*p* <0.001). In addition, there were significant differences in the immune subtypes of ASB16-AS1 in BRCA (*p* <0.001), LIHC (*p* =0.001), LUAD (*p* =0.008), and UCEC (*p* =0.021), which partially explains the prognostic role of ASB16-AS1 in different cancer types (Figure 6d).

### Identification of the relationship between lncRNA ASB16-AS1 and oncogenic signal pathways

**Figure 7.**
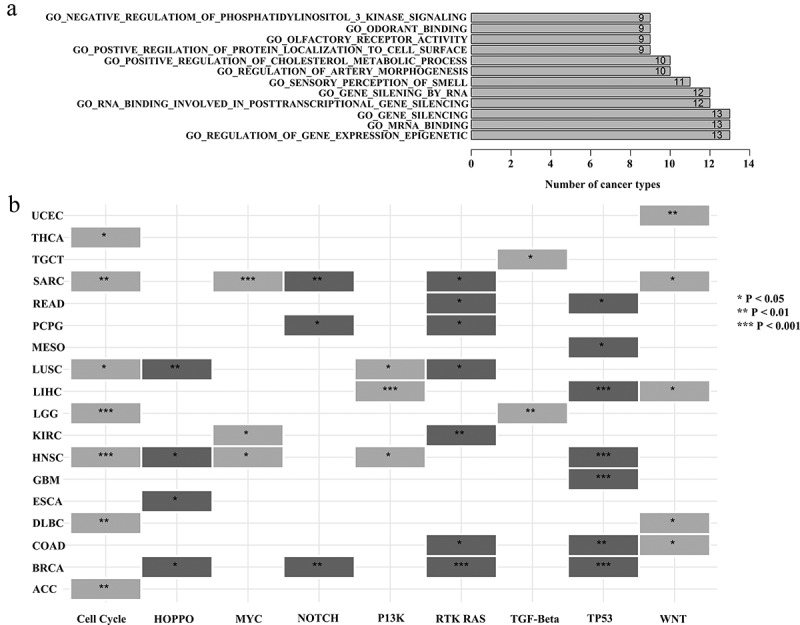
**Identification of the relationship between ASB16-AS1 and oncogenic signal pathways**. (a) The relationship between ASB16-AS1 and GO pathway in TCGA cancer analyzed by GSEA. (b) The relationship between ASB16-AS1 expression and alterations in 10 common oncogenic signaling pathways

Oncogenic signal pathways and the genome have complex mutual leases [21]. In order to further study the potential functions of ASB16-AS1, GSEA was performed and analyzed. GSEA analysis results showed that ASB16-AS1 affected over 10 immune-related signal pathways in multiple cancer types (such as, UVM, THYM, PRAD and ESCA). ASB16-AS1 affected ‘negative regulation of phosphatidylinositol 3 kinase sinnaling’, ‘odorant binding’, ‘olfactory receptor activity’, ‘positive regulation of protein localization of cell surface’, ‘positive regulation of cholesterol metabolic process’, ‘regulation of artery morphogenesis’, ‘sensory perceptioni of smell’, ‘gene silencing by RNA’, ‘RNA binding involved in posttranscriptional gene silencing’, ‘gene silencing’, ‘mRNA binding’ and ‘regulation of gene expression epigenetic’ in more 9 cancer types (Figure 7a). The spectrum of alterations in 10 common oncogenic signaling pathways among 33 cancer types in TCGA were obtained from the previous study [22]. In this study, among the 7 cancer types, ASB16-AS1 expression in the Cell cycle, RTK RAS and TP3 signaling pathway altered group was significantly compared with the non-alterations group. Among the 5 cancer types, ASB16-AS1 expression in the WNT signaling pathway altered group was significantly. Among the 4 cancer types, ASB16-AS1 expression in the HIPPO and MYC signaling pathway altered group was significantly (Figure 7b). In general, ASB16-AS1 has the potential to interact with multiple signaling pathways to exert carcinogenic effects.

### Identification of the relationship between lncRNA ASB16-AS1 and radiomics

**Figure 8.**
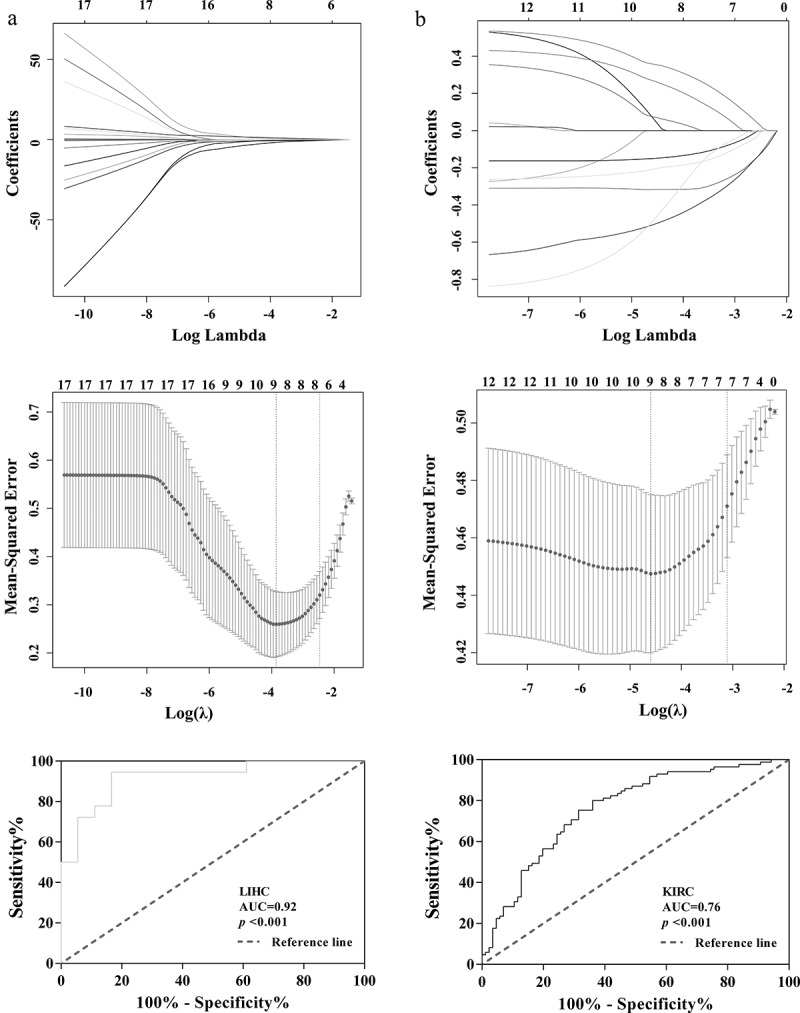
**Identification of the relationship between lncRNA ASB16-AS1 and**radiomics. ASB16-AS1 expression was significantly positively correlated with many radiomics features types. InIn LIHC, ASB16-AS1 expression was more correlated with radiomics features. The radiomics models identified the high and low expression of ASB16-AS1 (LIHC, AUC =0.92; KIRC, AUC =0.76) had good value

The possibility of radiomics to obtain tumor genome changes noninvasively has become a current research boom. ASB16-AS1 had significant positive correlation with multiple types of feature types. In LIHC, ASB16-AS1 and 1 Ipris (r =0.37), 61 CoLIAGe2D (r, 0.02–0.50), 15 Gabors (r, 0.14–0.40), wavelet (r, 0.23–0.33); and 2 WLLBP (r, 0.33–0.34) were significantly positively correlated. In KIRC, ASB16-AS1 and 24 original (r, 0.15–0.21), 7 Ipris (r, 0.15–0.23), 165 CoLIAGe2D (r, 0.001–0.23), 34 Gabors (r, 0.002–0.28), 78 wavelet (r, 0.05–0.21); and 1 WLLBP (r =0.17) were significantly positively correlated. It was not difficult to find that compared with KIRC, ASB16-AS1 was more related to radiomics in LIHC. CoLIAGe and Ipris features reflected the heterogeneity and difference in different tumors; Wavelets and Gabor features reflected tumor edge invasion information to a certain extent, which mean that ASB16-AS1 expression may had a certain parallel relationship with image information. We tried to divide ASB16-AS1 expression into high and low expression groups to find the differential expression features (Mann-Whitney U test, *p* <0.001). Then, the least absolute shrinkage and selection operator was used to further identify the features and corresponding weight coefficients of the radiomics models. Finally, the radiomics scores were obtained by linearly multiplying the feature values (LIHC, 9 features; KIRC, 9 features). The results showed that radiomics had a good identification value (LIHC, AUC =0.92; KIRC, AUC =0.76) (Figure 8). The relationship between radiomics and the genome still needs further exploration and verification.

## Discussion

In this study, we identified the expression and prognostic potential of lncRNA ASB16-AS1 molecular characteristics in more than 10,000 cancer samples from 33 cancer types, and deeply interpreted the relationship between its expression and prognosis, and performed expression verification in LIHC. In addition, our work provided the relationship between ASB16-AS1 expression and immune infiltration, potential evaluation markers of immunotherapy. Finally, we described a comprehensive view of the potential functional pathways of ASB16-AS1, and conducted a preliminary exploration of the relationship between the ASB16-AS1 expression and artificial intelligence. Our pan-cancer analysis showed that lncRNA ASAB16-AS1 was not only a potential prognostic marker, but also may played a role in immune infiltration and tumor immunology.

In recent years, multi-platform technological innovation has promoted a comprehensive interpretation of the transcription landscape of lncRNAs, and lncRNAs have been shown to be important regulators involved in biological processes [23]. To the best of our knowledge, ASB16-AS1 was first reported in the pubmed search engine in 2018 as a new functional gene of bone density variation [24]. After that, high ASB16-AS1 expression has been confirmed to be related to the tumor progression of glioma, non-small cell lung cancer, renal clear cell carcinoma, hepatocellular carcinoma, cervical cancer, esophageal cancer and gastric cancer [25–27]. On the basis of the above-mentioned high-quality studies, it was reasonable that ASB16-AS1 expression may affect the survival of tumor cells by deteriorating tumor cells. On this basis, we took LIHC as an example and verified through in vitro assays that ASB16-AS1 promoted tumor progression by affecting tumor cell proliferation or invasion. At the same time, our TCGA pan-cancer analysis results were similar to the above reports. It is still necessary to point out that although most cancer types are highly expressed in current reports and our analysis, the modes of action of ASB16-AS1 expression in different tumors still depend on the situation. For example, ASB16-AS1 was highly expressed in PAAD and represented a good prognosis. In this study, we confirmed the prognostic value of ASB16-AS1 in 33 cancer types. ASB16-AS1 high expression represented poor prognosis of ACC, COAD, KIRC, LIHC and PRAD. We further found that the up-regulation of ASB16-AS1 expression also affected the clinical stage of patients, and the expression was different in different immune subtypes.

Increasingly, lncRNAs have been confirmed by more and more high-quality studies as key regulators of gene expression in the immune system along with the heat of tumor immunotherapy [28]. Previous studies have mostly focused on the identification and prognostic value of immune-related lncRNAs. For example, the characteristics of immune-related lncRNAs assessed the potential response of immune checkpoint inhibitors in LIHC [29]. Immune-related lncRNAs were related to the infiltration of breast immune cell subtypes [30]. However, the overall transcription landscape for a single lncRNA and its relationship with immunity are still relatively small. Although the function of ASB16-AS1 in cancers has been in the preliminary exploration stage, only a few studies have been done, but ASB16-AS1 has not been fully studied in tumor immunology. In view of the excellent performance of ASB16-AS1 in tumor progression, its relationship with tumor immunology has aroused our keen interest.

Our study did find an association between ASB16-AS1 expression and the immune infiltration of multiple cancer types, even though their causality cannot be verified at present. As the tumor microenvironment contains numerous immune cells and stromal cells, it is often the place where immune cells infiltrate. The immune environment of different anatomical parts may have a reactive immune response to any anti-cancer therapy, which could be the cause of adverse results [31]. Our analysis showed that ASB16-AS1 expression was negatively correlated with immune cells and stromal cells of multiple cancer types. Surprisingly, ASB16-AS expression was positively correlated with the tumor purity of many cancer types (CESC, PAAD, UCEC, BLCA, BRCA, COAD, GBM, KIRP, LIHC, LUSC, PCPG, SARC and THCA), and only negatively correlated with TGCT. Tumor purity plays an important role in tumor growth, progression or drug resistance. For example, stromal cells can promote tumor growth and affected tumor treatment response [32], while immune cells such as tumor infiltrating cytotoxic T lymphocytes may inhibit tumor growth [33]. ASB16-AS1 expression has different relationships with tumor purity in different cancer types, which indicates that ASB16-AS has differences in carcinogenesis, progression, and therapeutic effects.

In addition, we further found that ASB16-AS1 expression was associated to different levels of immune infiltration in cancer types through a variety of immune cell content assessment methods. Immune cell content evaluation has differences, and multiple evaluation methods are mutually verified to effectively judge the relationship between ASB16-AS1 and immune cell subtypes [34,35]. Many immune cell subtypes are believed to play an important regulatory role in the development and treatment of cancers. For example, T cell CD4+ may hindered the patient’s response to immune checkpoint inhibitor blockade [36], and  T cells CD8+ had been confirmed it was a potential target marker for PD-L1 [37]. Our analysis results showed that ASB16-AS1 was positively correlated with B cells, T cells CD4+ and T cells CD8+ in most cancer types, and negatively correlated with dendritic cells, macrophages and neutrophils in some cancer types, especially neutrophils. T cells regulatory (Tregs) maintain immune balance by suppressing immune response through various multi-step contact-dependent and independent mechanisms, and have become a regulatory factor of concern for immunotherapy [38]. We further found that in most cancer types, ASB16-AS1 was positively correlated with T cells regulation (Tregs), especially in TGCT and KIRC. In summary, these findings indicate that ASB16-AS1 plays an important role in the recruitment and functional regulation of cancer immune infiltrating cells, which may ultimately affect the treatment and prognosis of patients.

Finally, we interpreted the relationship between ASB16-AS1 and some characteristics. First, immune checkpoint therapeutics use antibodies to destroy immune regulatory checkpoints and release preexisting anti-tumor immune responses, such as PD-L1 inhibitor [39]. The results of our analysis showed that ASB16-AS1 and PD-L1 inhibitor was significantly related in a variety of cancer types, and further analyzed the evaluation of ASB16-AS1 and potential immune effect evaluation molecules, such as TMB and MSI. The analysis results showed that the relationships between AB16-AS1 and them were not very strong. Secondly, the perturbation of the oncogenic signaling pathways affect the function of genes. In this study, unfortunately, no classical functional pathways were found through GESA. However, through analysis with common oncogenic signaling pathways, it was found that patients with changes in RTK RAS, Cell cycle and TP53 pathways had higher ASB16-AS levels in a variety of cancer types. Finally, radiomics artificial intelligence technology visualizes image information. Although it has not been proven that radiomics can predict non-coding RNA, more and more breakthroughs in radiomics technology have shown its possibility [40]. Our analysis results showed that radiomics features were significantly correlated with ASB16-AS1 expression in LIHC. We still believe that the radiomics is expected to reach the prediction of genome association analysis in the future.

Although we integrate transcriptome and phenotype data from multiple databases, the limitations of this study still exist. First of all, there were certain differences between microarray and sequencing data processing methods, which led to biases in the assessment of immune cell levels. Secondly, regarding contradiction in different LIHC database sources, it was found that the roles of ASB16-AS1 need to be further verified. Third, in this study, only the expression and prognosis of ASB16-AS1 were analyzed by bioinformatics, and the expression was only verified by in vitro experiments, and the roles of ASB16-AS1 were not further clarified at the mechanism level. Fourth, although ASB16-AS1 is found to be related to immune infiltration, the modes of action of the two have not been confirmed through specific experiments, which depends on future prospective studies to explain. Finally, there was no direct evidence for the connection between radiomics and genome, which is expected to be verified in the future.

## Conclusions

In summary, we explained the transcriptional expression, prognostic potential, relationship with tumor immune infiltration, and various potential functional pathways of lncRNA ASB16-AS1 from multi-omics and multi-platform perspectives. In addition, we further verified ASB16-AS1 transcriptional expression of LIHC through big data, and confirmed that ASB16-AS1 promoted the proliferation and invasion in LIHC by function assays. At present, ASB16-AS1 was still in the initial stage of exploration. These results confirmed the importance of ASB16-AS1 expression in the prognosis and treatment of cancers.

## Data Availability

The data sets generated and/or analyzed by the current study can be obtained from the corresponding author upon reasonable request.

## Highlights

(1) ASB16-AS1 was highly expressed in multiple cancer types, and it has been verified in large samples of liver hepatocellular carcinoma.

(2) High ASB16-AS1 expression showed poor OS and PFI in multiple cancer types.

(3) High ASB16-AS1 expression promoted the proliferation, migration and invasion of HCC cells.

(4) ASB16-AS1 expression was related to immune infiltration in multi-angle analysis.
